# *Helichrysum italicum* (Roth) G. Don and *Helichrysum arenarium* (L.) Moench Infusion Consumption Affects the Inflammatory Status and the Composition of Human Gut Microbiota in Patients with Traits of Metabolic Syndrome: A Randomized Comparative Study

**DOI:** 10.3390/foods11203277

**Published:** 2022-10-20

**Authors:** Ana Petelin, Karin Šik Novak, Matjaž Hladnik, Dunja Bandelj, Alenka Baruca Arbeiter, Katja Kramberger, Saša Kenig, Zala Jenko Pražnikar

**Affiliations:** 1 Faculty of Health Sciences, University of Primorska, Polje 42, 6310 Izola, Slovenia; 2 Faculty of Mathematics, Natural Sciences and Information Technologies, University of Primorska, Glagoljaška 8, 6000 Koper, Slovenia

**Keywords:** *Helichrysum italicum*, *Helichrysum arenarium*, metabolic syndrome, inflammation, gut permeability, gut microbiota

## Abstract

*Helichrysum italicum* (Roth) G. Don (HI) and *Helichrysum arenarium* (L.) Moench (HA) are rich in polyphenols and their infusions have beneficial effects for patients with metabolic syndrome. To investigate whether these effects are mediated by the gut microbiota, we analysed the effects of daily consumption of HI or HA infusion on the composition of gut microbiota, inflammatory status, and zonulin, a marker of gut barrier permeability. The study was a randomized, double-blind comparative trial. Thirty participants were randomly assigned to two groups and received either HA or HI tea filter bags, each containing 1 g of dried plant material, for daily consumption lasting 4 weeks. The results show that consumption of both infusions resulted in a reduction of some genera belonging to Firmicutes and in a slight but significant reduction in Shannon diversity index. Consumption of HI infusion significantly reduced serum levels of proinflammatory markers and zonulin alongside with the observed trend of Proteobacteria reduction. It can therefore be concluded that the HI and HA infusions could act as prebiotics and thus improve the intestinal environment. In addition, HI infusion has a positive impact on microbial dysbiosis and gut barrier dysfunction that occur in obesity and metabolic syndrome.

## 1. Introduction

Obesity and increased adiposity are characterized by high body mass index and excessive fat accumulation and are the main causes of metabolic syndrome (MS) [1]. Compared to lean individuals, the adipose tissue of obese individuals secretes high levels of pro-inflammatory cytokines such as TNF-α, IL-1β, and IL-6, induces systemic inflammation and insulin resistance [2], and increases the risk of certain types of chronic diseases such as non-alcoholic fatty liver disease, cardiovascular disease, and type 2 diabetes mellitus, thereby reducing quality of life and life expectancy [3]. Therefore, it is crucial to find effective treatments for obesity.

The causes of obesity and overweight are complex and may include genetic, dietary, and environmental factors [3]. One of the most important interfaces between the external world and the internal human environment is the gastrointestinal tract. As a natural process indispensable for health, a complex microbial consortium forms in the human gastrointestinal tract immediately after birth [4]. A reciprocal relationship with the gut microbiota is critical for human health, as the microbiota not only maintains the integrity of the intestinal epithelium [5] and regulates the digestion, synthesis, and absorption of many nutrients and metabolites [6], but also contributes to the regulation of organs outside the gut, such as liver and brain, and the immune system [7,8,9,10]. Obesity and MS are often associated with gut bacterial dysbiosis, with recent evidence revealing a significant difference in the gut microbiota between lean and obese individuals [11,12]. In addition to healthy faecal microbiota transplants administered to individuals with illnesses, diet composition and nutritional status have been found to be among the modifiable factors controlling gut microbiota [13]. Changes in macronutrient composition have been shown to contribute substantially [14]. In addition, prebiotics obtained from fruits and vegetables can regulate host lipid metabolism and glucose homeostasis by reversing gut dysbiosis in obese mice [15]. Therefore, targeting the gut microbiota appears to be a promising approach to improve metabolic health associated with obesity.

Infusions of *Helichrysum italicum* (Roth) G. Don (also called immortelle or everlasting, abbreviated as HI) and *Helichrysum arenarium* (L.) Moench (sandy everlasting, abbreviated as HA) with plethora of polyphenols, such as hydroxycinnamic acids and pyrones, hydroxybenzoic acids, arzanol and flavonoids [16,17] exert beneficial effects for patients with obesity and MS [18,19]. We have recently shown that the intake of HI or HA has favourable but different effects on the traits of MS [19], where HI was more efficient in reducing body mass and fat mass, whereas HA efficiently reduced total and LDL cholesterol levels. In addition, a single ingestion of HI infusion increased fat oxidation and resting energy expenditure in healthy male participants [18]. These effects could be attributed to direct effects of bioactive compounds on peripheral organs, but there are other possible mechanisms. We have shown that HI can markedly affect cells of intestinal wall; in human primary colon fibroblasts, treatment with HI infusion altered the expression of genes involved in cytoskeleton rearrangement and cell growth, while pathway analysis revealed the importance of interleukin signaling [20]. We therefore proposed that the main mode of action of HI is the indirect prevention of diseases due to the improvement or maintenance of barrier integrity [20]. In this context, the role of microbiota is also very important, and it is possible that the effects of HI and HA on the components of MS are mediated by microbiota in addition to the direct effects of bioactive compounds on the intestinal wall cells and other internal organs. There are several bioactive compounds present in HI and HA that could potentially interact with the gut microbiome. Polyphenols, are a class of prebiotics that are resistant to host’s digestion and can be fermented by gut microorganisms [21]. They are thought to have a bidirectional relationship with the gut microbiota by influencing its composition and enabling it to further metabolise polyphenols and generate bioactive compounds [22]. Polyphenols’ health benefits are mainly associated with their antioxidant and anti-inflammatory properties [21].

In the present study, we therefore investigated the effects of daily consumption of one cup of HI or HA infusion over four weeks on the composition of the gut microbiota, inflammatory status, and zonulin, a marker of gut barrier permeability, which could help us explain the beneficial effects previously observed in participants with at least two traits of MS.

## 2. Materials and Methods

### 2.1. Study Participants

**Figure 1:**
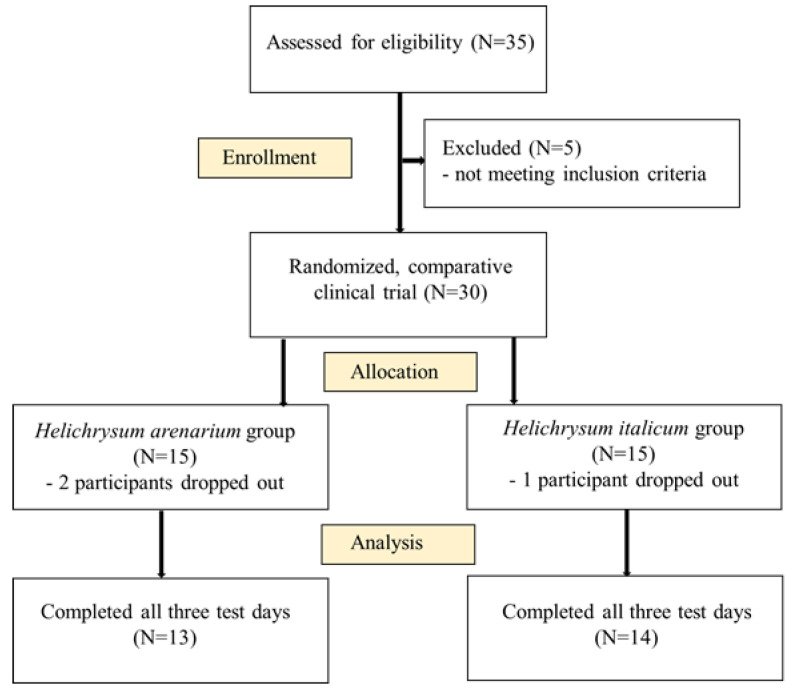
CONSORT flow diagram.

Thirty-five participants were invited to participate in the current study. Five participants did not meet the inclusion criteria, so the final study included thirty adults with at least two traits of metabolic syndrome. Sample size was calculated based on primary outcome as described [19], where inclusion and exclusion criteria are also reported. CONSORT flow diagram is shown in Figure 1. The final sample size was 19 women and 8 men. Written informed consent was obtained from all participants in the clinical trial. The protocol was approved by the National Ethics Committee (No. 0120-557/2017/4) and registered on Clinicaltrials.gov (NCT04866628).

### 2.2. Study Protocol

The study was a double-blind randomized comparative trial. Thirty participants were randomly assigned to two groups—the *Helichrysum arenarium* (HA) group or the *Helichrysum italicum* (HI) group. Randomization with stratification was performed as described [19]. The study was conducted at the University of Primorska, Faculty of Health Sciences, between May and July 2021. The entire study consisted of a 4-week administration phase (intervention) in which subjects in both groups were prescribed to drink 200 mL of either HA or HI tea every evening 2 h after dinner; and a 2-week follow-up phase without any supplementation. Participants were asked not to change their normal diet and physical activity, except for the prescribed tea consumption. All participants refrained from taking antibiotics and other dietary supplements or medications. An experienced dietitian collected a list of all ingested foods and beverages. Dietary data was analyzed using the Open Platform for Clinical Nutrition (OPEN) accessible through the website http://opkp.si/. A researcher contacted the participants weakly to ensure adherence to the supplementation protocol.

Subjects visited the faculty at baseline, after 4 weeks of the intervention period and after a follow-up period. They completed questionnaires about the frequency of gastrointestinal symptoms and fasting blood samples for biochemical analysis were collected at the same time points. Patients were instructed to collect stool samples for microbiota analysis one day before or on the day of the measurements. Stool collection tubes and ice-packs were provided in advance. Participants collected a pea-size sample and froze it immediately. The samples were then stored at −80 °C until further use.

### 2.3. Test Beverages and Dosages

The plant material used to prepare the test beverages was obtained from two *Helichrysum* species: dried plant material of commercially available *Helichrysum arenarium* (L.) Moench tea, purchased from Flora Ltd., Rogatec, Slovenia, and *Helichrysum italicum* (Roth) G. Don plants harvested from commercial plantation in Dragonja, Slovenian Istria (45°27′05″ N 13°41′31″ E). Herbarium specimen with the accession number Hia-UP20 is stored at the University of Primorska, Faculty of mathematics, natural sciences and information technologies. Subjects were given either HA or HI tea filter bags each containing 1 g of dried plant material to consume daily for 4 weeks. They were instructed to prepare hot water extracts (i.e., infusions) just before consumption by immersing the tea filter bags in hot water (200 mL, 100 °C) for 10 min. Both test beverages were similar in color and taste and in the size and shape of the filter bags to ensure that they were indistinguishable. 

Chemical composition of both beverages was analyzed with HPLC-MS and was discussed in our previous papers [16,17,18]. Briefly, several phenolic classes were identified. The majority of the compounds identified in the HI beverage belonged to a caffeoylquinic acids subclass, arzanol derivatives and other pyrones. In comparison to HI, HA infusion was richer in flavonoids, in particular flavanones, whereas flavonols, flavones, hydroxycinnamic acids, pyrons, and cumarins were not detected [17].

### 2.4. Inflammatory Parameters

Venous blood samples were collected in 6 mL vacuum test tubes (Beckton-Dickinson, Rutherford, USA) after an overnight fast. Serum was immediately separated by a 10 min centrifugation at 2000× *g*, frozen, and stored at −80 °C until subsequent analysis. Serum concentrations of interleukin-6 (IL-6), interleukin-1β (IL-1β), interleukin-33 (IL-33), MCP-1 and zonulin were determined in duplicate on a microplate reader (Tecan, Männedorf, Switzerland) using human ELISA Kits for IL-6, IL-1β, IL-33, MCP-1 (BioVendor, Brno, Czech Republic) and zonulin (MyBiosource, San Diego, CA USA). Assay sensitivity was 0.32 pg/mL for IL-6, 0.4 pg/mL for IL-1β, 0.9 pg/mL for IL-33, 2.3 pg/mL for MCP-1 and 0.5 ng/mL for zonulin. Assays interassay and intraassay CVs were typically <7% for interleukins and <12% for zonulin.

### 2.5. Gastrointestinal Symptoms

The participants were also asked to complete a self-administered scale to evaluate the presence and frequency or severity of bowel symptoms during the preceding week. The self-administered questionnaire consisted of the following 6 items: nausea/vomiting, bloating, borborygmi, abdominal pain, flatulence, and burning or epigastric pain. Each symptom was scored with respect to either the frequency [0—never, 1—hardly ever, 2—sometimes, and 4—quite a few or many times] or intensity (0—none, 1—light, 2—moderate, and 3—severe) of the symptom. The overall score was calculated by multiple severity and frequency scores.

### 2.6. Stool Sample Analysis

A fresh stool sample from each subject was stored in stool collection tubes at −80 °C until analysis. For the assessment of the microbial community composition, DNA was extracted from the frozen faecal samples (1–2 g) using the commercial QIAamp DNA Stool Mini Kit (Qiagen N. V., Venlo, The Netherlands) following the manufacturer’s instructions and stored at −80 °C for further analysis. Amplification of V4 16S rRNA was performed with 515F (modified by [23] and 806R (modified by [24]). Primer 806R was elongated with the sequence of P1 adapter and 515F primer was elongated with the sequence of linker, barcode and adapter at their 5′ end in order to produce amplicons compatible for sequencing with Ion S5TM System (Thermo Fisher Scientific, Waltham, MA USA). Each sample was amplified with unique barcode in triplicates to reduce PCR bias. Negative control was performed with unique barcode as well. PCR reaction and temperature profile were set as described in Earth Microbiome Project (www.earthmicrobiome.org) [25].

PCR products were checked on agarose gel and equal volume of pooled triplicates were joined in a final library. Final library was cleaned with Agencourt AMPure XP magnetic beads (Beckman Coulter) using bead-to-DNA ratio of 0.7:1. Quality of DNA library and DNA concentration was determined on Agilent 2100 Bioanalyzer using High Sensitivity DNA Assay kit (Agilent Technologies, Santa Clara, CA USA). Ion 530™ chip with DNA library and Ion S5™ calibration standard was prepared with Ion OneTouch™ 2 system using the kit Ion 520™ & Ion 530™ Kit-OT2 and sequencing was performed on Ion S5™ System (Thermo Fisher Scientific, Waltham, MA USA). 

Reads were analysed with QIIME2 v.2021.8 [26]. Firstly, only sequences with forward and reverse primer were extracted with Cutadapt (qiime cutadapt trim-single method) and primers were trimmed [27]. DADA2 [28] (qiime dada2 denois-pyro plugin) was used for denoising and determination of amplicon sequence variants (ASVs) with no additional trimming. 

Taxonomy classification was made with feature classifier plugin [29]. The naive Bayes classifier [30] was trained with amplicon region specific sequences extracted from SILVA reference database, version 138.1 with representative sequences at 99% identity [31]. Reference database was prepared with RESCRIPt [32]. ASVs not assigned to the Bacteria kingdom were removed. 

### 2.7. Microbiota Data Analysis

Statistical analysis regarding gut microbiota was performed in R using different packages. Alpha diversity was evaluated with Shannon index, estimated with DivNet method (argument “tuning” was set to “careful”) implemented in DivNet R package [33] and richness with breakaway method as a part of breakaway R package [34]. Differences in Shannon index and richness between samples from the same subjects at the baseline and after intervention, as well as between samples at baseline from different groups were evaluated with betta random (where subjects were treated as a random effect) and betta functions of the breakaway package [35]. Type of intervention and groups formed on the basis of body mass index (BMI) were considered as fixed effects (subjects were overweight with 25 kg/m^2^ < BMI < 29.9 kg/m^2^ or obese with BMI > 30.0 kg/m^2^). Labels of taxa, which were not determined at the genus level, or were identified as uncultured were modified with the addition of first known higher taxonomy classification level. Modified taxonomy table was collapsed to genus level before beta diversity assessment with Aitchison distances, i.e., clr- (centered log-ratio) transformation of counts was performed with R package microbiome [36] and transformed counts were subjected to the PCA using ordinate function of phyloseq R package [37].

Core microbiota, i.e., taxa with 75% prevalence among all samples and average relative abundance of 0.5% were determined on dataset, resampled to an even depth of 25,000 reads per sample and median, mean and standard deviation was presented. MaAsLin2 R package [38] was used to identify differentially abundant taxa on the phylum and genus level between the two sampling points (baseline and after intervention) within HA and HI groups. Subjects were treated as random effect and BMI groups (overweight and obese) and time of sampling as fixed effects. Taxa observed in at least 50% of samples in HA or HI group were included in the analysis. For the differential abundance analysis table with relative abundance values was used as an input for Maaslin2 function with the following default settings: normalization = “TSS”, transform = “LOG” and analysis_method = “LM”, indicating linear model with TSS (total sum scaling) normalization and log transformation. Same parameters (excluding random effect) were used to compare subjects from different interventions (HA and HI): at the baseline, and after supplementation period. Taxa were treated as significant if q value was below 0.250.code.

### 2.8. Statistics

Statistical analysis was performed using SPSS version 23.0 (IBM Corp., Armonk, NY USA). The normality of variables was tested by the Shapiro–Wilk test. Data are presented as the mean value with standard deviation or median value with minimum and maximum or as the percentage. The effects of the interventions within each group were analyzed using a Student’s paired samples t-test or Wilcoxon signed-rank test, whereas comparison of mean changes between the 2 groups was analyzed using an independent t-test or Mann–Whitney U test. Moreover, the effects of the interventions were analyzed using an univariate analysis of covariance (ANCOVA) with the change at week 4 or follow-up from baseline as the dependent variable, adjusted to the corresponding values at baseline, and stratified for several variables (age, sex, and body fat and biochemical variables at baseline). *p* values < 0.05 were considered statistically significant.

## 3. Results

### 3.1. Baseline Characteristics of Subjects

Basal characteristics of the subjects assigned to both groups were discussed in [19]. Groups (HA vs. HI) were not significantly different at baseline with respect to age, sex, smoking habits, blood pressure, and anthropometry. In addition, energy intake and macronutrient composition of the diets did not differ between the two groups at baseline. None of the baseline differences between the groups were statistically significant indicating that the groups were well matched.

### 3.2. Effects of HI and HA Infusions on Inflammatory Markers and Zonulin

The measured inflammatory parameters and zonulin, a biomarker of intestinal barrier permeability, are summarized in Table 1. Baseline concentrations of inflammatory markers IL-6, IL-1β, IL-33, and zonulin were not significantly different between the two groups, whereas serum levels of MCP-1 at baseline were significantly higher in the HI group than in the HA group. The serum levels of zonulin significantly decreased after the HI-supplementation period (F = 5.561, *p* = 0.046). Regarding the inflammatory markers, in the HI group, paired t-test revealed a significant decrease in IL-6 levels and a significant increase in IL-33 level at week 4. Moreover, the same effect for IL-6 was also observed after adjustment for the described variables (ANCOVA model). Furthermore, the ANCOVA model revealed additional significant decreases in serum levels of IL-1β and MCP-1 at week 4. During follow-up, the observed effects were no longer significant.

On the other hand, in the HA group the paired t-test and ANCOVA model showed a significant decrease in the serum levels of IL-1β at week 4. At that point, there was no change in zonulin levels (F = 0.006, *p* = 0.940). A significant increase during follow-up was observed for IL-33 by the paired t-test and for MCP-1 by the ANCOVA model. 

Due to similar trends in the dynamics of inflammatory markers, no significant differences were observed in the changes between the two groups (Table 1).

### 3.3. Effects of HI and HA Infusions on Gut Microbiota

In the present study, the microbial profiles of 27 individuals at the baseline and after 4 weeks of nutrition supplementation were analyzed. To assess the impact of HI and HA infusions consumption on gut microbiome, we performed high-throughput sequencing of the 16S rRNA gene V4 regions of the faecal samples. Reads were denoised with DADA2 and in total 25,842 to 79,244 reads per sample (57018 in average) were retained in a final dataset.

**Figure 2:**
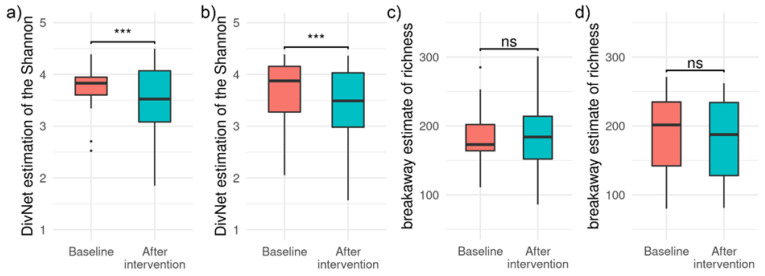
Distribution of estimated Shannon index for HA (**a**) and HI (**b**) groups and richness for HA (**c**) and HI (**d**) groups at baseline (red box plot) and after intervention (blue box plot). Significance bar indicates significant (*** *p* < 0.001) or non-significant (ns) differences.

The Shannon diversity index and richness calculated for each sample are shown in Figure 2. The Shannon index significantly decreased after the period of HA- and HI-supplementation (*p* < 0.001 in both cases), while no change was observed for richness (HA group: *p* = 0.487, HI group: *p* = 0.125). At baseline, Shannon index and richness did not differ between HA and HI group (Shannon index: *p* = 0.959, richness: *p* = 0.308).

**Figure 3:**
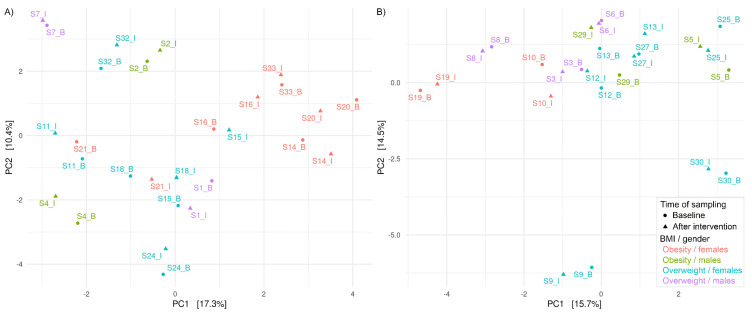
PCA plot with clr-transformed counts on genus level based on the samples from HI group (**A**) and HA (**B**) group. Sample names are composed of the letter S and number of each subject followed by B (sampled at baseline) or I (sampled after intervention). Legend applies to both PCA plots. BMI, body mass index.

PCA plots with clr-transformed counts at the genus level for the HA and HI groups (Figure 3) indicate that samples at the baseline and after the intervention cannot be discriminated based on the treatments, whereas similarity between samples from the same participants can be observed.

Core microbiota, composed of taxa present in more than 75% of the samples, and with an average relative abundance of at least 0.5% were identified to get insight into the changes of the most frequently observed taxa. For each group, HI and HA, at baseline and intervention median, mean and standard deviation values were calculated (Table 2). The major bacterial communities at the phylum level which were detected in the microbiota of all participants were Bacteroidota, Firmicutes and Proteobacteria. At baseline, Bacteroidota was the predominant phylum in both groups followed by Firmicutes and Proteobacteria. The most abundant genera also belonged to Bacteroidota phyla (*Bacteroides* and *Alistipes*) and Firmicutes phyla (*Faecalibacterium*, *Lachnospiraceae NK4A136*, *Clostridia UCG-014*, *Roseburia* and *Agathobacter*). In general, there were no significant differences between the two groups at the baseline level, and neither at the end of the intervention (Table 2).

At the phylum level, the statistical analyses revealed decrease in the relative abundance of Proteobacteria from 8.79 ± 8.14 to 4.62 ± 3.56 (*p* = 0.049, q = 0.287) after HI treatment; however, q-value is above the threshold of 0.250. Moreover, in both groups (HI and HA) a trend of increase in the relative abundance of Bacteroidota and decrease in the relative abundance of Firmicutes and Proteobacteria was observed, but the changes were not significant.

**Figure 4:**
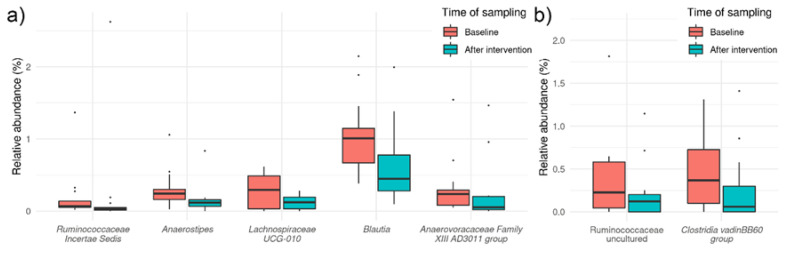
Box plot showing bacterial abundance before and after intervention in *Helichrysum arenarium* (HA) group (**a**) and in *Helichrysum italicum* (HI) group (**b**). Five taxa at the genus level with *p* < 0.05 and q < 0.250 were considered significantly different between the time points in HA group and only two taxa at the genus level with *p* < 0.05 and q < 0.250 were considered significantly different between the time points in HI group.

At the genus level, the analyses demonstrated that the HA treatment for 4 weeks significantly decreased the relative abundance of *Lachnospiraceae UCG-010* (*p* = 0.003, q = 0.0.189), *Blautia* (*p* = 0.005, q = 0.189), *Anaerostipes* (*p* = 0.002, q = 0.189), *Ruminococcaceae Incertae sedis* (*p* = 0.05, q = 0.189) and *Anaerovoracaceae* Family XIII AD3011 group (*p* = 0.006, q = 0.189) (Figure 4a). *Christensenellaceae R-7* (*p* = 0.034, q = 0.617), *Faecalibacterium* (*p* = 0.030, q = 0.617), *Ruminococcaceae UCG-005* (*p* = 0.045, q = 0.617), *Parabacteroides* (*p* = 0.044, q = 0.617) and *Lachnospiraceae NK4A136* (*p* = 0.046, q = 0.617) were among taxa with *p* value below 0.05, but above q-value threshold for significance.

On the other hand, the analyses showed that the HI treatment for 4 weeks significantly decreased the relative abundance of *Ruminococcaceae* uncultured (*p* = 0.005, q = 0.179) and *Clostridia vanidin BB60* group (*p* = 0.011, q = 0.231) (Figure 4b). Decrease in *Lachnospiraceae UCG-001* (*p* = 0.024, q = 0.261) can be mentioned although decrease is non-significant according to the q-value.

Due to the same trend in changes in microbiota composition during intervention in both groups, no significant differences between the groups were observed.

### 3.4. Effects of HI and HA Infusions on Gastrointestinal Symptoms

Table 3 represents the self-reported gastrointestinal symptoms at baseline, week 4 and during follow-up. Average gastrointestinal symptom scores were not significantly different between the two groups at any of the time points. At baseline, the highest scores (i.e., the most frequent) for digestive symptoms were for bloating and flatulence in both groups, while the lowest was for nausea. There were significant reductions in gastrointestinal symptoms, as after 4 weeks of supplementation with HA improvement in bloating (53.8%), borborygmi (53.8%), abdominal pain (46.2%), and flatulence (46.2%) was reported by the participants and there was even further improvement during follow-up (61.5%, 53.8%, 46.2%, 61.5%, respectively). In addition, after 4 weeks of supplementation with HA 38% of subjects reported improvements in burning or epigastric pain. On the other hand, less improvements in gastrointestinal symptoms were shown in HI group. Here, the improvements were observed in bloating (57.1%), flatulence (35.7%), and in burning or epigastric pain (42.9%), but only after 4 weeks of supplementation.

## 4. Discussion

HI and HA infusions have recently been associated with a number of health benefits related to metabolic disease [18,19]. Specifically, consumption of HI infusion had a beneficial effect on body weight, visceral, and total body fat, while HA infusion had a more favourable effect on serum glucose levels and lipid profile [19]. Accumulating evidence indicates that the gut influences metabolic health [39] and that polyphenol supplementation can influence the composition of the gut microbiota and thus metabolic health [40]. Therefore, the purpose of the present study was to explore the effect of HI and HA infusions on the gut microbiome and to test our hypothesis that bioactive compounds from *Helichrysum *sp*.* infusions alter it alongside with the reduced levels of inflammation in participants with traits of MS. Our results suggest that consumption of HI and HA infusions for 4 weeks alters the composition of the gut microbiota and reduces serum levels of inflammatory markers in overweight and obese individuals, but there are some differences in the activities of the two infusions.

HI infusion efficiently reduced gut permeability and inflammation. The two parameters are closely connected and related to obesity and MS. Our study demonstrates that daily intake of HI infusion for 4 weeks promotes a significant decrease in circulating levels of zonulin, and pro-inflammatory cytokines including IL-6, IL-1β, and MCP-1. On the other hand, infusion of HA had no effect on serum zonulin levels, and the effects on inflammatory markers were less prominent after 4-week intervention compared to HI. However, two weeks after both interventions, IL-1β was significantly decreased only in the HA group. The differences between HI or HA effects are probably related to their chemical profile. Arzanol, a phloroglucinol-α-pyrone, present in HI and HA, is known to inhibit the inflammatory transcription factor NFκB, whose activation releases IL-1β, IL-6, IL-8, and TNF-α [41]. Indeed, a decrease in IL-1β was observed in both groups. In addition to arzanol, hydroxycinnamic acid derivatives, especially chlorogenic acids, have shown numerus health benefits in the treatment of obesity, cardiovascular disease, type 2 diabetes mellitus, and MS [42,43]. HI infusion contains higher levels of chlorogenic acid and also has higher potential in reducing total body fat and visceral fat [19], all of which may be related to the reduced serum levels of inflammatory markers observed in the present study. In various experimental protocols, chlorogenic acid and its major metabolites such as ferulic acid, caffeic acid and isoferulic acid were detectable in the systemic circulation [44] and could directly affect various peripheral tissues. Furthermore, both infusions were able to induce an increase in IL-33 in agreement with our previous transcriptome analysis, where IL-33 was the major up-regulated gene in the primary colon cell line treated with HI infusion [20]. Although IL-33 can act as either pro- or anti-inflammatory cytokine, it was shown that administration of recombinant IL-33 in genetically obese diabetic (*ob/ob*) mice resulted in reduced adiposity, decreased fasting glucose, and improved glucose and insulin tolerance, suggesting a protective role of IL-33 in the development of adipose tissue inflammation during obesity [45].

However, molecular mechanism of HI infusion regarding inflammation is not completely understood. In our recently published paper, where colon fibroblasts were treated with HI infusion, biological processes related to hemostasis, wound healing, cytoskeletal rearrangement and epithelial development were proposed as the main pathways affected by HI [20]. Observed changes in global gene expression pointed to the activity of HI in regulating intestinal epithelial barrier and thereby protecting underlying tissues from harsh external environments. Therefore, the observed reduced inflammation could also be mediated by the ability of HI to maintain intestinal epithelial barrier. Reduced level of serum zonulin observed in the present study supports this hypothesis.

It is also possible that inflammation is reduced due to the change in gut microbiota composition. Diet is one of the main factors in shaping gut microbiota composition, and when diet enriched with more anti-inflammatory components such as polyphenol rich plant foods, it exerts antioxidant, anti-inflammatory, and immunomodulatory effects [46]. Studies indicate a two-way relationship between polyphenols and gut microbiota. Gut microbiota metabolizes polyphenols, and polyphenols and their metabolites modulate gut microbiota by inhibiting pathogenic bacteria [47]. In the present study, the supplementation with HI and HA infusions increased the overall anti-inflammatory potential of subjects’ otherwise un-changed diet. Even though the overall structure of the participants’ gut microbiota and richness remained stable during the 4-week interventions, both infusions induced a slight but significant reduction of gut community diversity, as observed with estimated Shannon index. The same phenomenon was observed before in a short-term high-fibre diet which induced a slight drop in α-diversity [48]. In contrast with our results where decreased inflammation alongside with lower diversity was observed, loss of biodiversity within the gut has been linked to an increasing number of conditions such as gastrointestinal diseases and obesity-associated inflammatory characteristics, while increased diversity has been associated with increased health [49]. A slight drop in α-diversity obtained in the current study might be a result of reduced proportion of particular taxa, since richness was not significantly reduced and may reflect the “shock effect” of a relatively rapid change in the spectrum of incoming polyphenols and other bioactive compounds, which may transiently disrupt the ecology of the gut community. However, HI infusion also induced a reduction of the relative abundance of Proteobacteria (*p* < 0.05, q = 0.287). Although q-value is above the threshold 0.250, this important finding may partially explain the observed reduced systemic inflammation in the HI group as it is known that Proteobacteria form a minor part of the healthy gut microbiota, but when they disproportionately expand, this can lead to unspecific inflammation. Proteobacteria are often overrepresented in several intestinal and extraintestinal diseases, usually with an inflammatory phenotype [50] and are positively associated with obesity [8]. Class gamma-Proteobacteria carry LPS molecules that are potent triggers of inflammatory responses. In enterocytes, LPS induces the release of interleukin 8—a key proinflammatory chemokine, which alters tight junctions and leads to impaired epithelial integrity [51]. It is therefore possible that the observed zonulin decrease and reduced inflammation are due to the reduced abundance of Proteobacteria.

In addition to Proteobacteria, Bacteroidota and Firmicutes are the most important groups involved in colon metabolism of undigested food remnants, including dietary fibre and polyphenols [52]. A healthy gut microbiota has been typically characterized by members of the Bacteroidota and Firmicutes phyla, and their genera are believed to be the main responsible bacteria for positive biodiversity in the human gut [53], as their balanced abundances and metabolite production protect the intestinal tract, help digestion and modulate the host innate immune system [54]. In the current study, significant decrease in the relative abundance of some genera belonging to Firmicutes was observed after the consumption of either HI or HA infusion, confirming the modulatory effect of both infusions on the intestinal microbiota composition. It has been already suggested that Firmicutes are repressed to a larger extent by polyphenols and their metabolites, thus tilting the balance in favor of Bacteroidota in the human gut [55]. Moreover, in an obese adult population, the ratio Firmicutes/Bacteriodota was higher compared to lean participants [56]; therefore, our findings imply that HA and HI infusions could contribute to weight loss though this mechanism. This hypothesis is further supported by the previous reports on anti-obesity herbal medicines and functional foods that can modify the gut microbiota by increasing the richness of the phylum Bacteroidota, and of genera *Akkermansia*, *Lactobacillus*, and *Bacteroides*, and by reducing that of the phylum Firmicutes and the Firmicutes/Bacteroidota ratio [57].

At the genera level, HI infusion significantly reduced the relative abundance of *Ruminococcaceae* uncultured and affected *Lachnospiraceae* *UCG-001*, which were also found to correlate with increased fat mass and BMI [58,59]. In addition, HI infusion significantly reduced short chain fatty acids (SCFAs)-producing bacteria *Clostridiales vadin BB60* [60] which have been recently shown to be inversely correlated with obesity, dyslipidaemia, and insulin resistance in the mice model [61] and with BMI, weight, and waist circumference in women [62].

In comparison with HI, HA infusion significantly reduced *Lachnospiraceae UCG-010*, *Blautia* and *Ruminococcaceae Incertae sedis* genus. Although members of Lachnospiraceae are among the main producers of SCFAs, different taxa of Lachnospiraceae are also associated with different intra- and extraintestinal diseases. *Blautia* are known to stimulate cytokines secretion by host cells [63] and are positively related to long-chain triglycerides, impaired glucose metabolism, and type 1 diabetes [64]. Similar to our study, *Blautia* has also been reduced in response to black tea and red wine grape extract in an in vitro gut microbial ecosystem [65]. It is important to remark that Lachnospiraceae (in particular *Blautia*) as well as *Ruminococcaceae Incertae sedis* play a key role in the metabolism of undigested carbohydrates [66]. Despite the beneficial effects concerning SCFAs production from saccharolytic metabolism [67], carbohydrate digestion by gut microbiota contributes to increasing the energy derived from the diet, and thus, affecting fasting blood glucose levels. Therefore, significant depletion in *Blautia*, *Lachnospiraceae UCG-010* and *Ruminococcaceae Incertae sedis* after HA supplementation could explain observed metabolic improvements in our subjects [19]. Further, HA supplementation resulted in decrease in *Christensenellaceae* *R-7* and *Faecalibacterium* (*p* < 0.05, q > 0.250) which are considered as biomarkers of intestinal and host wellness hence their reduction may explain the fact that HI has a stronger anti-inflammatory effect. *Faecalibacterium* has the ability to produce anti-inflammatory molecules [68,69] and has been found to be consistently reduced in inflammatory diseases [70]. Furthermore, the inverse association between *Christensenellaceae* and BMI has been reported as one of the strongest links between the microbiome and metabolic diseases [71]. Loss of bacteria that produce the anti-inflammatory, barrier-strengthening molecule butyrate could lead to a loss of protection against epithelial inflammation and gut barrier disruption. Indeed, in the present study HA infusion did not have an effect on zonulin, and its effects on inflammatory parameters were weaker compared to HI, probably due to stronger reduction of relative abundance of specific SCFAs-producing genera belonging to Firmicutes. On the other hand, the relative abundance of *Prevotella 9* (from 5.42 ± 11.28 to 8.35 ± 17.34) and *Parabacteroides* (from 1.11 ± 0.51 to 1.80 ± 1.07; *p* < 0.05, q > 0.250) increased in HA group, but not significantly. Nevertheless, *Prevotella* species were shown to be positively associated with propionate production that has an important role in preventing weight gain by reducing serum cholesterol and decreasing hepatic lipogenesis [72]. Indeed, reduced total cholesterol levels, TAG, AST and ALT in HA group were observed in our subjects and discussed in previous paper [19]. The overall changes reiterate the complexity of the gut microbiome and the fact that changes can rarely be classified as simply “good” or “bad”. Furthermore, the magnitude of the effect on gut microbiota during these short-term dietary interventions is, as expected, relatively modest in relation to the interindividual variability of the microbial profile, as it is known that in general only extreme dietary shifts elicit more pronounced effects [73]. Additional changes could be expected if the intervention was longer. Here, this was not tested, since we focused only on the underlaying mechanism which could explain the important health-related changes in lipid profile and anthropometric measures observed previously [19].

Since we have demonstrated that HI and HA alter the composition of the gut microbiota and due to the known relationship between gastrointestinal symptoms and gut microbiota dysbiosis [74,75], we were additionally interested in whether herbal infusions have the potential to improve these symptoms. Both infusions were effective, confirming the commonly reported traditional use of *Helichrysum* infusions for digestive complaints [76]. There was a significant improvement in most gastrointestinal symptoms (bloating, borborygmi, abdominal pain, and flatulence) after 4 weeks of HA supplementation and during follow-up. Nausea was the only symptom that did not improve during this intervention, probably due to the low frequency of this symptom in our study population. Gastrointestinal symptoms occur mainly due to carbohydrate fermentation followed by substantial gas production [74]. The improvement in gastrointestinal symptoms by consumption of herbal infusions might be related to a reduced abundance of bacteria releasing hydrogen, carbon dioxide, methane, and other gases produced by fermentation. *Blautia*, for example, that was found to produce very high amounts of hydrogen was reduced after HA intervention [77]. In addition, the improvement could also be related to decreased serum levels of IL-1β during the intervention, indeed it was reported that elevated IL-1β levels were associated with abdominal bloating and loose stools in irritable bowel patients [78]. In both groups IL-1β levels significantly decreased after 4 weeks of intervention and in HA group, IL-1β remained significantly lower also during follow-up and similar fluctuations were observed for the gastrointestinal symptoms. It should be noted that the sole experience of participating in a trial could have an impact on health-related outcomes, especially on self-reported gastrointestinal symptoms. Due to the absence of a placebo group, this possibility cannot be completely excluded.

Overall, our results show that HI and HA infusions in the prevention and treatment of obesity may be closely related to their role in regulating the gut microbiota. Supplementation with HI and HA altered its composition, confirming their prebiotic activities. Additionally, this study provides new insights into the potential metabolic effects of HI and HA infusions. HI significantly, but transiently, reduced inflammation and improved gut permeability at the end of the 4-week intervention. On the other hand, positive effects of HA were observed during the follow-up, especially the improvement in gastrointestinal symptoms and decreased levels of IL-1β. The results suggest that both infusions could be used for reducing chronic low-grade inflammation associated with obesity. In the case of HI, where the observed benefits were lost after the end of intervention, regular consumption is recommended. Whether HA or HI -based treatment approaches could have an impact on acute inflammation and acute gastrointestinal problems requires further investigation. The study design is such that it is easy to reproduce in real life, since the required daily intake of the infusion was relatively low. The efficiency of such protocols was also implied by Khairudin et al. [79], who have recently shown that low doses of tea (*Camellia sinensis*) can more efficiently increase the diversity of the gut microbiota in a short period of time, compared with higher doses of tea administered over a longer period. However, the observed “shock effect”, although slight, suggests that longer interventions may have additional benefits and should also be tested. The obtained results are thus important for public health and should help to develop an effective strategy to mitigate the growing burden of MS. Nevertheless, the cross-talk between metabolites and specific alteration of intestinal bacteria in host physiology, as well as the precise contributing compounds present in herbal infusions should be subjects of future studies.

## Figures and Tables

**Table 1:** Inflammatory markers before and after intervention.

	*Helichrysum arenarium* Group (N = 13)			*Helichrysum italicum* Group (N = 14)		
	Baseline	Week 4	Follow-Up	Baseline	Week 4	Follow-Up
IL-6 (pg/mL)	2.06 ± 2.60	1.74 ± 2.43	1.67 ± 1.86	4.13 ± 5.34	2.74 ± 3.14 ^a,c^	4.12 ± 4.48
IL-1ß (pg/mL)	1.06 ± 0.59	0.61 ± 0.47 ^a^	0.59 ± 0.56 ^c^	2.02 ± 1.97	1.81 ± 2.56 ^c^	1.58 ± 2.99
MCP-1 (pg/mL)	70.66 ± 28.89	90.91 ± 41.38	92.53 ± 39.44 ^c^	101.53 ± 46.52 ^b^	97.32 ± 45.99 ^c^	96.90 ± 64.24
IL-33 (pg/mL)	25.06 ± 9.81	32.65 ± 30.99	36.74 ± 11.93 ^a^	26.02 ± 14.37	40.86 ± 33.50 ^a^	28.94 ± 14.61
Zonulin (ng/mL)	6.06 ± 3.04	6.93 ± 3.34	6.95 ± 3.86	6.19 ± 1.91	5.65 ± 1.53 ^c^	6.68 ± 3.28

Abbreviations: IL-6 = interleukin 6; IL-1ß = interleukin 1 beta; IL-33 = interleukin 33; MCP-1 = monocyte chemoattractant protein-1. Values are expressed as means ± SD. ^a^ *p*-value denotes significant (*p* < 0.05) difference within group when compared with the initial values using a Student’s paired samples t-test or Wilcoxon signed-rank test. ^b^ *p* value denotes significant (*p* < 0.05) difference between the two groups (*Helichrysum italicum* vs. *Helichrysum arenarium*) at a given time point. ^c^ *p*-value denotes significant (*p* < 0.05) effects of the intervention analysed using a univariate analysis of covariance (ANCOVA) with the change at week 4 or follow-up from baseline as the dependent variable, adjusted to the corresponding values at baseline, and stratified for age, sex, and body fat.

**Table 2:** Core microbiota composition at the Phylum and Genus level in subjects with traits of metabolic syndrome before and after intervention.

	*Helichrysum arenarium* Group (N = 13); Median/Mean ± SD		*Helichrysum italicum* Group (N = 14); Median/Mean ± SD	
	Baseline	Week 4	Baseline	Week 4
Phylum Bacteroidota	50.41/49.39 ± 18.12	62.59/55.82 ± 21.3	49.18/52.05 ± 19.13	64.86/59.15 ± 19.12
Genera				
*Bacteroides*	36.17/33.56 ± 22.11	25.98/38.8 ± 27.01	27.85/35.82 ± 23.98	46.13/43 ± 24.96
*Alistipes*	4.39/4.41 ± 2.67	2.76/3.72 ± 2.78	4.71/4.88 ± 3.92	4.60/4.74 ± 3.22
*Parabacteroides*	0.93/1.11 ± 0.51	1.86/1.80 ± 1.07	0.98/1.47 ± 1.06	1.28/1.32 ± 0.68
*Barnesiella*	0.43/1.09 ± 2.09	0.49/0.55 ± 0.53	0.35/0.96 ± 1.25	0.32/0.74 ± 0.85
*Odoribacter*	0.45/0.47 ± 0.35	0.53/0.47 ± 0.3	0.40/0.64 ± 0.66	0.63/0.68 ± 0.44
Phylum Firmicutes	41.47/41.67 ± 14.82	33.43/35.57 ± 19.18	35.68/37.06 ± 18.78	29.65/33.66 ± 18
Genera				
*Faecalibacterium*	5.52/6.58 ± 3.73	3.58/5.17 ± 4.15	9.35/9.74 ± 6.86	6.39/8.28 ± 6.27
*Lachnospiraceae NK4A136*	1.61/2.30 ± 2.84	2.46/2.32 ± 2.27	1.34/1.57 ± 1.32	1.39/1.99 ± 2.22
*Clostridia UCG-014*	1.48/2.74 ± 3.49	1.38/1.8 ± 1.57	0.70/1.12 ± 1.4	0.56/1.62 ± 3.43
*Roseburia*	1.48/1.96 ± 1.77	1.07/1.9 ± 2.48	0.82/1.48 ± 1.53	1.64/1.7 ± 1.39
*Agathobacter*	2.99/2.96 ± 2.43	1.66/2.08 ± 2.48	0.82/0.95 ± 0.74	0.71/0.96 ± 1.03
*Subdoligranulum*	1.17/1.21 ± 0.76	1.62/1.47 ± 0.88	0.95/1.33 ± 1.07	0.85/1.39 ± 2.21
*Lachnospiraceae* unidentified	1.13/1.41 ± 1.38	0.67/0.86 ± 0.55	1.19/1.48 ± 1.17	1.25/1.62 ± 1.59
*Oscillospiraceae UCG-002*	0.99/1.62 ± 1.76	1.14/1.11 ± 0.83	0.94/1.25 ± 1.09	0.85/1.09 ± 1.05
*Christensenellaceae R-7*	0.36/1.17 ± 2.21	0.19/1.39 ± 3.65	0.57/1.06 ± 1.36	0.28/0.67 ± 0.94
*Coprococcus*	0.82/0.74 ± 0.33	0.79/0.81 ± 0.53	0.67/1.10 ± 1.29	0.69/0.91 ± 0.74
*Ruminococcus*	0.53/0.75 ± 0.85	0.32/0.53 ± 0.66	0.42/1.12 ± 1.68	0.52/1.08 ± 1.43
*Blautia*	0.99/1.05 ± 0.54	0.52/0.64 ± 0.58 ^a^	0.79/0.86 ± 0.56	0.53/0.6 ± 0.38
*Lachnosphira*	0.43/0.63 ± 0.96	0.38/1.03 ± 2.2	0.50/0.52 ± 0.4	0.15/0.26 ± 0.29
[Eubacterium] *coprost. group*	0.26/0.53 ± 0.84	0.14/0.38 ± 0.55	0.42/0.67 ± 0.66	0.22/0.65 ± 1.07
*Ruminococcaceae CAG-352*	0.22/0.47 ± 0.68	0.17/0.24 ± 0.27	0.42/0.94 ± 1.98	0.39/0.46 ± 0.47
*Clostridia vanidin BB60*	0.50/0.81 ± 1.11	0.29/0.52 ± 0.6	0.38/0.53 ± 0.62	0.06/0.27 ± 0.43 ^a^
Lachnospiraceae *UCG-004*	0.47/0.45 ± 0.33	0.26/0.35 ± 0.28	0.47/0.48 ± 0.39	0.48/0.73 ± 0.82
Phylum Proteobacteria	6.05/6.74 ± 5.67	5.40/5.68 ± 3.21	5.43/8.79 ± 8.14	3.53/4.62 ± 3.56
Genera				
*Sutterella*	3.06/2.75 ± 1.81	2.57/2.92 ± 1.59	1.97/2.7 ± 2.5	1.06/1.74 ± 1.44

Values (relative abundances of phyla and genera) are expressed as median value/means ± SD. Taxa of the genera with relative abundance >0.50% of total reads and with a prevalence of >75% are included. ^a^ q-value denotes significant (q < 0.250) difference when compared to the initial value within the same group.

**Table 3:** Frequency of gastrointestinal symptoms before and after intervention.

	*Helichrysum arenarium* Group (N = 13)			*Helichrysum italicum* Group (N = 14)		
Scores	Baseline	Week 4	Follow-up	Baseline	Week 4	Follow-up
Nausea	1.15 ± 0.38	1.00 ± 0.00	1.00 ± 0.00	1.14 ± 0.54	1.50 ± 0.94	1.29 ± 0.61
Bloating	2.62 ± 1.04	1.69 ± 0.86 ^a^	1.54 ± 0.52 ^a^	2.36 ± 1.08	1.71 ± 0.83 ^a^	1.93 ± 0.92
Borborygmi	2.00 ± 0.91	1.38 ± 0.65 ^a^	1.38 ± 0.51 ^a^	2.07 ± 0.62	1.86 ± 0.95	1.79 ± 0.80
Abdominal pain	1.69 ± 0.95	1.08 ± 0.28 ^a^	1.00 ± 0.00 ^a^	1.36 ± 0.63	1.43 ± 0.85	1.43 ± 0.76
Flatulence	2.61 ± 1.12	1.85 ± 0.90 ^a^	1.85 ± 0.38 ^a^	2.36 ± 0.93	2.00 ± 0.78 ^a^	2.00 ± 0.68
Burning or epigastric pain	1.69 ± 1.03	1.15 ± 0.56 ^a^	1.31 ± 0.48	1.93 ± 0.92	1.43 ± 0.76 ^a^	1.50 ± 0.76

Frequency of gastrointestinal symptoms was evaluated by a questionnaire from 1 (never), 2 (hardly ever), 3 (sometimes) to 4 (quite a few or many times). Values are expressed as means ± SD. ^a^ *p* value denotes significant (*p* < 0.05) difference within group when compared with the initial value using a Wilcoxon signed-rank test.

## Data Availability

The V4 16S rRNA amplicon sequencing data associated with this study have been deposited in the NCBI Sequence Read Archive under the BioProject accession number PRJNA888462. Other data presented in this study are available on request from the corresponding author.
